# An Ode to Texas Doctors

**Published:** 1898-04

**Authors:** 

**Affiliations:** M. D., G. T. O. W., T. D. I. I., A. T. C. T., Houston, Texas


### Correspondence.

### An Ode to Texas Doctors.

LEGISLATORS WILL PLEASE JOIN IN THE CHORUS.
*Tune of John Brown's Body.*
SHORT METRE.

O Brothers! go and git a pestle,
And fiat some pizen—PILLS,
And lam 'em straight into the gozzle
Of law-makers. Till they—WILL
Repeal the obnoxious "doctor's tax,"
Or grant us leave to—KILL
Representatives, etcetera,—with an axe
If they beat their doctor's—BILL.

BACILLUS ICTEROIDES, M. D.,
G. T. O. W., T. D. I. I., A. T. C. T.,
Houston, Texas.

I.E.—Give them one week—to do it in—after they come together.—[Ed.]

